# A novel stepwise catheter ablation method of the mitral isthmus for persistent atrial fibrillation: efficacy and reproducibility

**DOI:** 10.1186/s12872-023-03490-7

**Published:** 2023-09-15

**Authors:** Jingchao Li, Shihua Cui, Huihui Song, Luqian Cui, Haijia Yu, Yingjie Chu, Shujuan Dong

**Affiliations:** 1 Department of Cardiology, Henan Provincial People’s Hospital, Zhengzhou, China; 2 Dalian Medical University, Dalian, China

**Keywords:** Persistent atrial fibrillation, Mitral isthmus ablation, Catheter ablation, Ethanol infusion of the vein of Marshall

## Abstract

**Background:**

Ethanol infusion of the vein of Marshall (EI-VOM) has been widely used to facilitate mitral isthmus (MI) ablation. According to the literature, the success rate of achieving a bidirectional conduction block across the MI ranges from 51 to 96%, with no standardized strategy or method available for cardiac electrophysiologists.

**Objectives:**

This study aimed to introduce and evaluate a novel ablation method of MI.

**Methods:**

Consecutive patients with persistent atrial fibrillation (PeAF) that underwent catheter ablation were included. The MI ablation procedure followed a stepwise approach. In step 1, ethanol infusion of the vein of Marshall (EI-VOM) was performed. In step 2, a “V-shape” endocardial linear ablation connecting the left inferior pulmonary vein (LIPV) to mitral annulus (MA) was performed. In step 3, earliest activation sites(EASs) near the ablation line were identified using activation mapping followed by reinforced ablation. In step 4, precise epicardial ablation was performed, with the catheter introduced into the coronary sinus(CS) to target key ablation targets (KATs).

**Results:**

135 patients with PeAF underwent catheter ablation with the stepwise ablation method adopted in 119 cases. Bidirectional conduction blocks were achieved in 117 patients (98.3%). The block rates of every step were 0%, 58.0%, 44.0%, and 92.9%, and the cumulative block rates for the four steps were 0%, 58.0%, 76.5%, and 98.3%, respectively. No patient experienced fatal complications.

**Conclusions:**

Our novel stepwise catheter ablation method for MI yielded a high bidirectional block rate with high reproducibility.

## What is new?

- A novel ablation method of the mitral isthmus (MI) was introduced, which exhibited a high bidirectional block rate.
- The ablation method involved Ethanol infusion of the vein of Marshall (EI-VOM), “V-shape” linear ablation, and endocardial plus epicardial ablation of key ablation targets (KATs), which can be abbreviated as “MVK”.
- The novel stepwise ablation method is easy to conduct and demonstrates reproducibility in achieving block rates when performed by different electrophysiologists in various centers.

## Introduction

Although pulmonary vein isolation (PVI) remains the cornerstone of catheter ablation for atrial fibrillation (AF), it may not be sufficient to sustain sinus rhythm in persistent AF (PeAF) patients [1]. In recent years, more extensive ablations have been advocated to improve the success rate of PeAF catheter ablation, including linear ablation, complex fractioned electrograms, and so on [2–4].

The mitral isthmus (MI), involving the left inferior pulmonary vein (LIPV), Left atrial appendage (LAA), coronary sinus (CS), ligament of Marshall (LOM), and mitral annulus (MA), is a critical structure for minimizing the recurrence of PeAF [5, 6]. However, achieving bidirectional conduction block poses technical challenges due to its anatomical complexity and epicardial electrical connections [7]. One potential approach is to target the vein of Marshall (VOM), which traverses the epicardial aspect of the MI. It is now understood that ethanol infusion of the VOM (EI-VOM) achieves a conduction block by directly damaging the atrial myocardium through retrograde ethanol infusion. Several studies have demonstrated the effectiveness of EI-VOM in facilitating MI ablation [8–10]. Although EI-VOM has gained acceptance and improved the success rate of MI block, different strategies and methods have been employed, resulting in variable success rates ranging from 51–96% [8–11]. Moreover, these approaches have not displayed consistent reproducibility among cardiac electrophysiologists.

Based on our experience, we described a novel stepwise ablation method in the present study, which involved EI-VOM, “V-shape” linear ablation, and endocardial plus epicardial ablation of key ablation targets (KATs), referred to as “MVK” for simplicity.

## Method

### Population and study design

From March 2022 to March 2023, consecutive PeAF patients undergoing catheter ablation at Henan Provincial People’s Hospital were enrolled. All patients underwent cardiac contrast-enhanced computed tomography or transesophageal echocardiography to rule out left atrial thrombosis. Moreover, antiarrhythmic drugs (amiodarone or others) were discontinued for a minimum of five half-lives before the ablation procedure.

### Preoperative preparation and intra-procedural setting

All procedures were performed under general anesthesia by anesthetists. A steerable decapolar catheter (DecaNAV; Biosense Webster, Irvine, USA) was used to construct the matrix and advanced into the CS. Two transseptal punctures were performed under X-ray and intracardiac echocardiographic guidance. A Pentaray catheter (Biosense Webster, Irvine, USA ) was used for constructing electroanatomical maps of pulmonary veins (PVs) and the left atrium (LA). A 56 porous irrigated-tip CF sensing catheter (Thermocool SmartTouch SF; Biosense Webster, Irvine, USA) was introduced into the LA for localization and ablation via the steerable sheath (VIZIGO; Biosense Webster, Irvine, USA).

The PeAF ablation strategy involved PVI, superior vena cava isolation (SVCI), and linear ablations of MI, cavotricuspid isthmus, and LA roof. If AF did not terminate after PVI, sinus rhythm (SR) was restored by transthoracic cardioversion. During SR, linear ablations were performed. The radiofrequency (RF) energy was delivered by an ablation index (AI)-guided high-power ablation strategy with 50 W except for the CS ablation of 25 W (irrigation flow 15 ml/min). Predefined VisiTag (Biosense Webster, Irvine, USA) settings (the lesion-tag size 2 mm, interlesion distance 4 mm, minimum time 3 s) were used to automatically display RF applications.

**Fig. 1:**
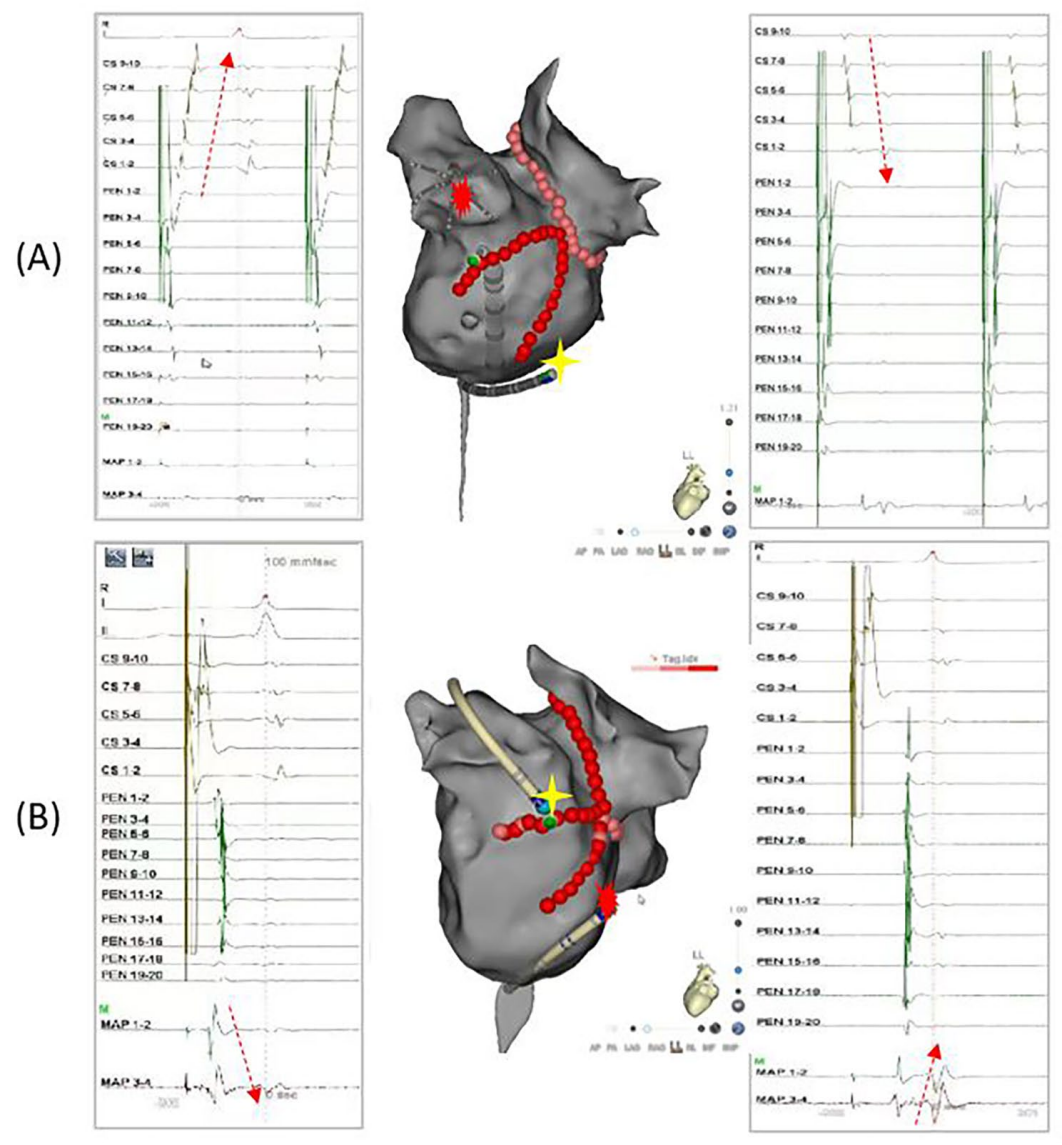
Endpoint of the mitral isthmus (MI) ablation. (**A**) Before ablation, the coronary sinus (CS) catheter recorded a CS1-2 to CS 9–10 activation sequence when pacing at the Left atrial appendage (LAA) by the Pentaray catheter (left). After ablation, the activation sequence was transformed into CS9-0 to CS1-2 (right); (**B**) Before ablation, the ablation catheter recorded a 1–2 to 3–4 activation sequence when pacing at the distal of the CS catheter (left). After ablation, the activation sequence was transformed into 3–4 to 1–2 (right)

The endpoint of the MI ablation was defined as the achievement of a bidirectional conduction block meeting the following protocol. When pacing was performed on one side of the block line, a distal-to-proximal activation sequence was observed on the other, demonstrating an activation detour (Fig. 1).

### Workflow of the novel stepwise ablation method

All MI ablation procedures followed the four steps below strictly.

#### Step 1

**Fig. 2:**
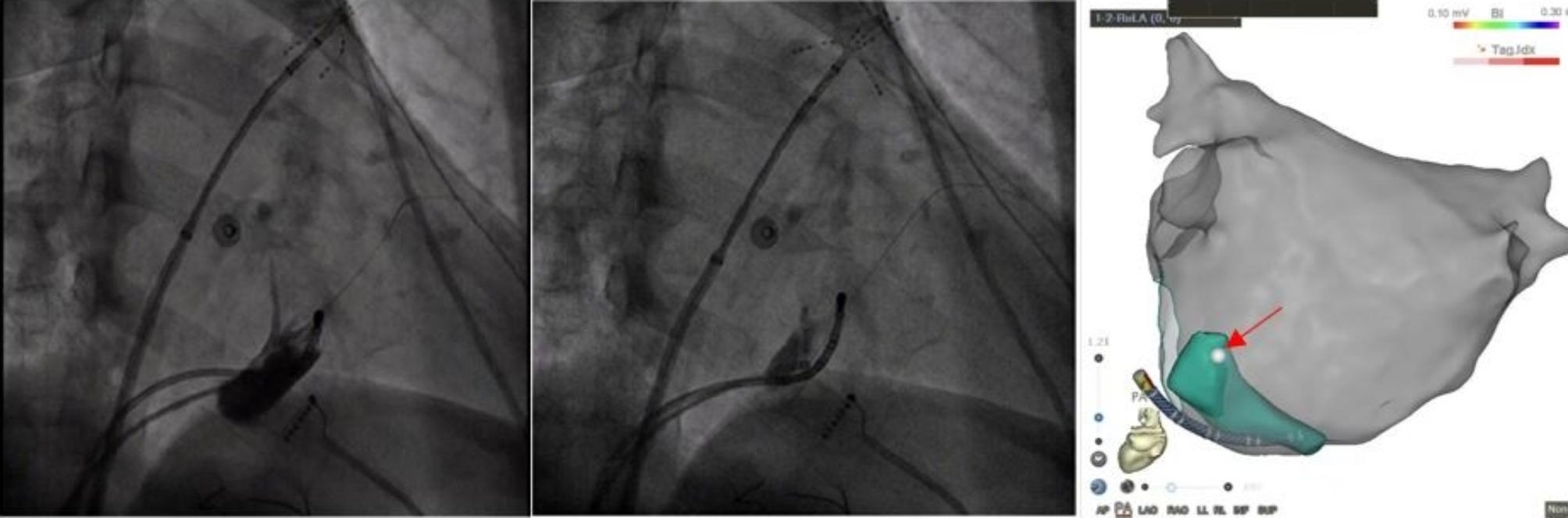
Ethanol infusion of the vein of Marshall (EI-VOM) and marked VOM ostium(red arrow)

EI-VOM was first performed. The coronary sinus was cannulated by a modified 6 F Judkins right guiding catheter (Judkins R4.0) inside the Swartz sheath (Abbott, Chicago, USA). CS venograms were performed to identify the presence of the VOM. If present, an over-the-wire angioplasty balloon (Emerge 1.5-2.5 mm x 6–8 mm; Boston Scientific) preloaded with guidewire was advanced into the proximal VOM. 5–8 ml 96% ethanol was injected into the VOM over 1 min. In addition, the ostium of VOM was marked on the reconstructed CS map. The conduction block was evaluated after completing PVI. If the endpoint was not achieved, step 2 was initiated. (Fig. 2)

#### Step 2

**Fig. 3:**
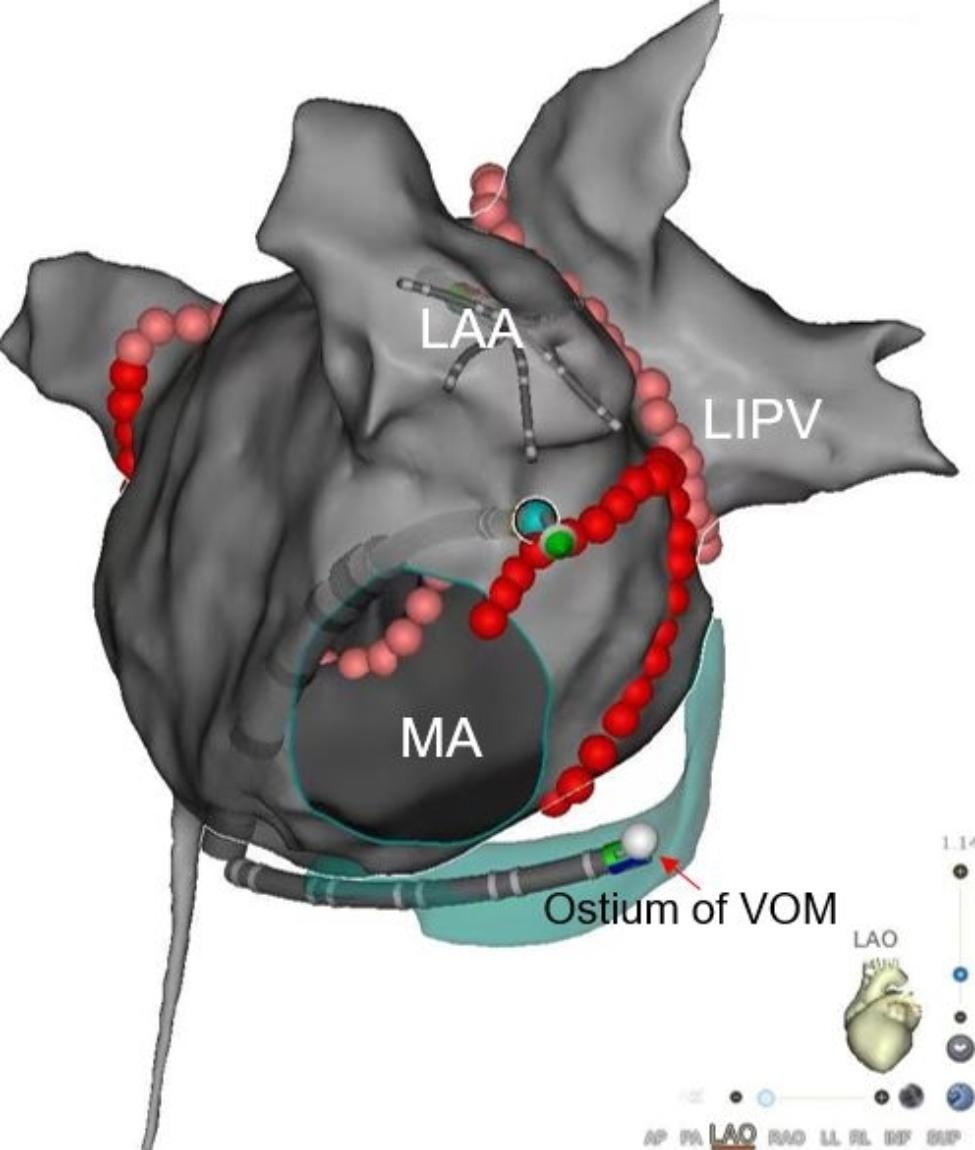
Endocardial “V-shape” linear ablation of the mitral isthmus (MI). The upper ablation line was closed to the root of the Left atrial appendage(LAA) from the 1–2 o’clock point of the mitral annulus at the Left anterior oblique (LAO) 45º view to the left inferior pulmonary vein(LIPV), closing to the root of LAA. The lower ablation line coursed from the LIPV to the lower MA anatomically corresponding to the ostium of the vein of Marshall (VOM)

In this step, endocardial MI linear ablation was performed while pacing in the LAA with 500ms S1S1 using the PentaRay catheter, and the potentials of CS were recorded dynamically. The upper ablation line was created from the 1–2 o’clock ventricular lateral of MA (local potential: higher amplitude of the ventricular wave than that of atrium) at the Left anterior oblique(LAO) 45º view and extended towards the LIPV, closing in proximity to the root of the LAA. The lower ablation line was created from the LIPV to the lower ventricular lateral of MA, which corresponded to the ostium of VOM marked in step 1, typically located at the 5–6 o’clock position of MA. These two lines formed a “V-shape” linear ablation for the MI. Moreover, effective lesions (ELs) were identified and marked when the activation sequence changed or/and the conduction time was prolonged during ablating. Reinforced ablation was performed on those ELs when the ablation endpoint was not achieved or reconnection was discovered after 20 min of observation. If the endpoint was not reached, step 3 was initiated (Fig. 3).

#### Step 3

**Fig. 4:**
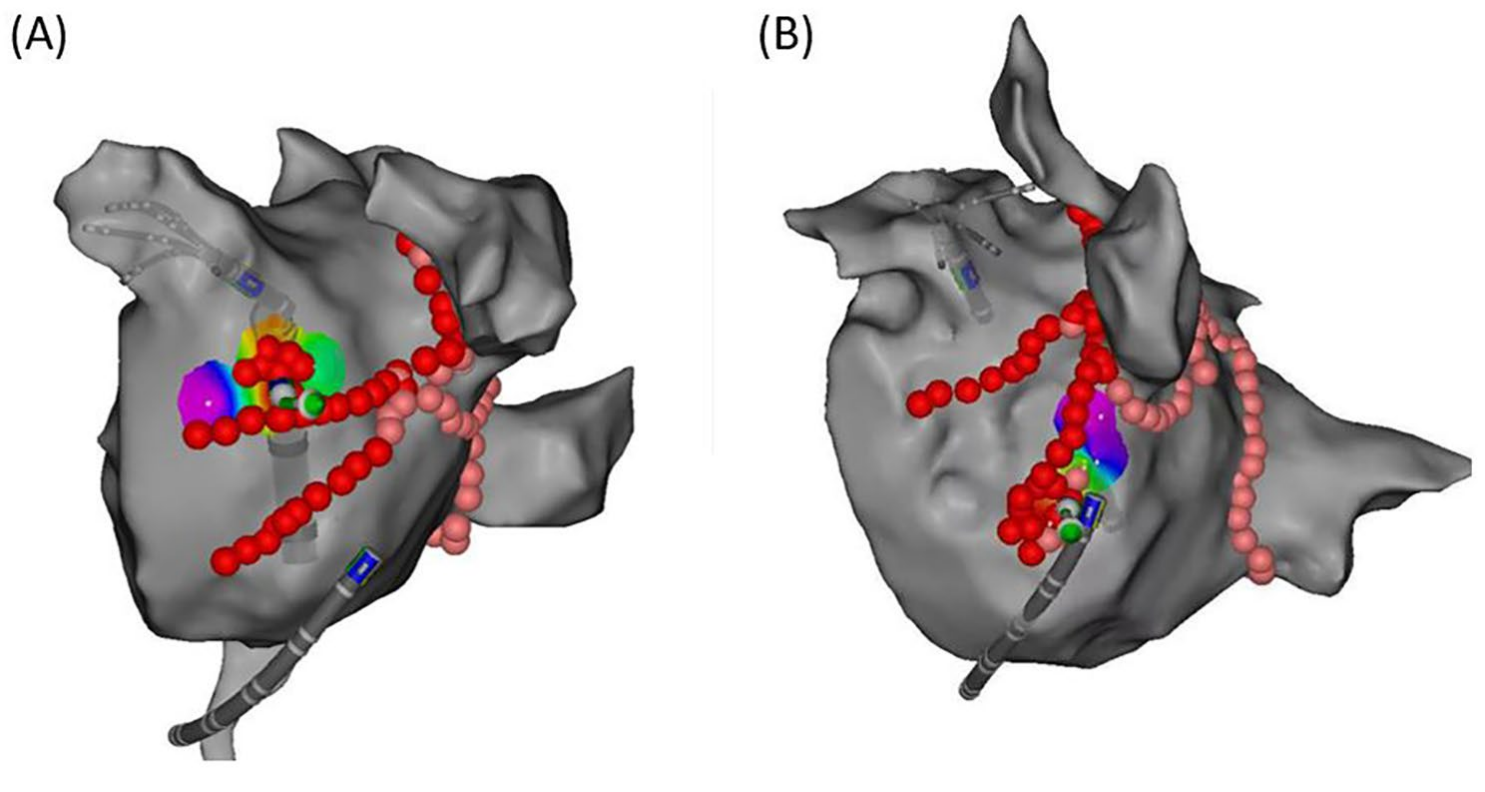
Activation mapping and reinforced ablation of the earliest activation sites (EASs). (**A**) The EASs around the upper line of the “V-shape” ablation were identified, and reinforced ablation was performed; (**B**) The EASs around the lower line were identified, and reinforced ablation was performed

**Fig. 5:**
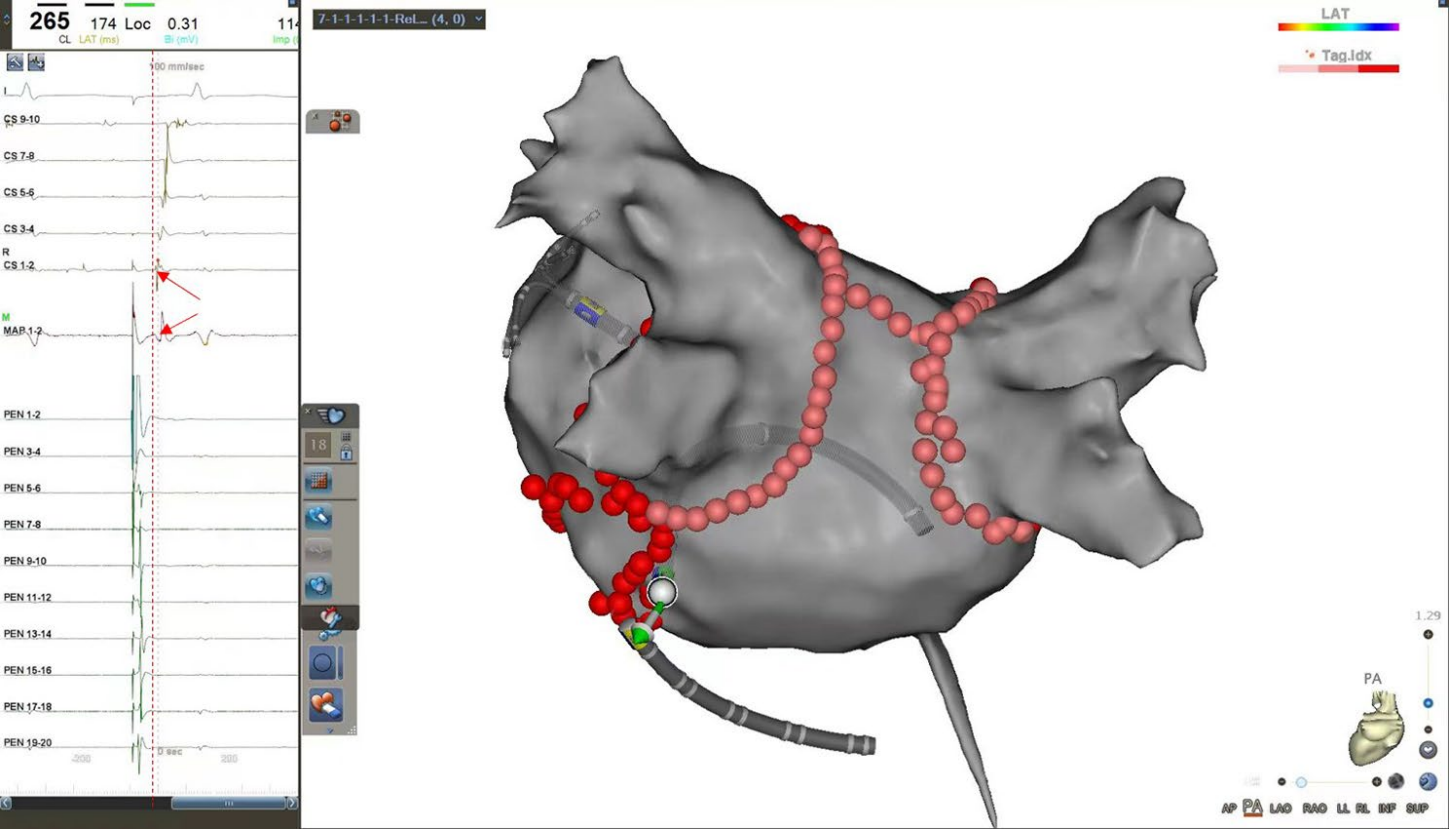
An earlier epicardial activation than the endocardium. activation sequence on CS1-2 (epicardium) was earlier than MAP1-2(endocardium) when ablating earliest activation sites (EASs).

Activation mapping was performed to identify the earliest activation sites around the two “V-shape” ablation lines. An ablation catheter was used for this mapping. During the distal of CS pacing, EASs around the upper line were identified, while the EASs around the lower line were identified during LAA pacing (Fig. 4). Then reinforced ablation targeting EASs was performed. Once the EASs were identified, reinforced ablation targeting these sites was performed. The location of the EASs was recorded and analyzed when a conduction block was achieved after ablation. Step 4 was initiated if an earlier epicardial activation than the endocardium was observed (Fig. 5) or the endpoint was not achieved after reinforced ablation.

#### Step 4

**Fig. 6:**
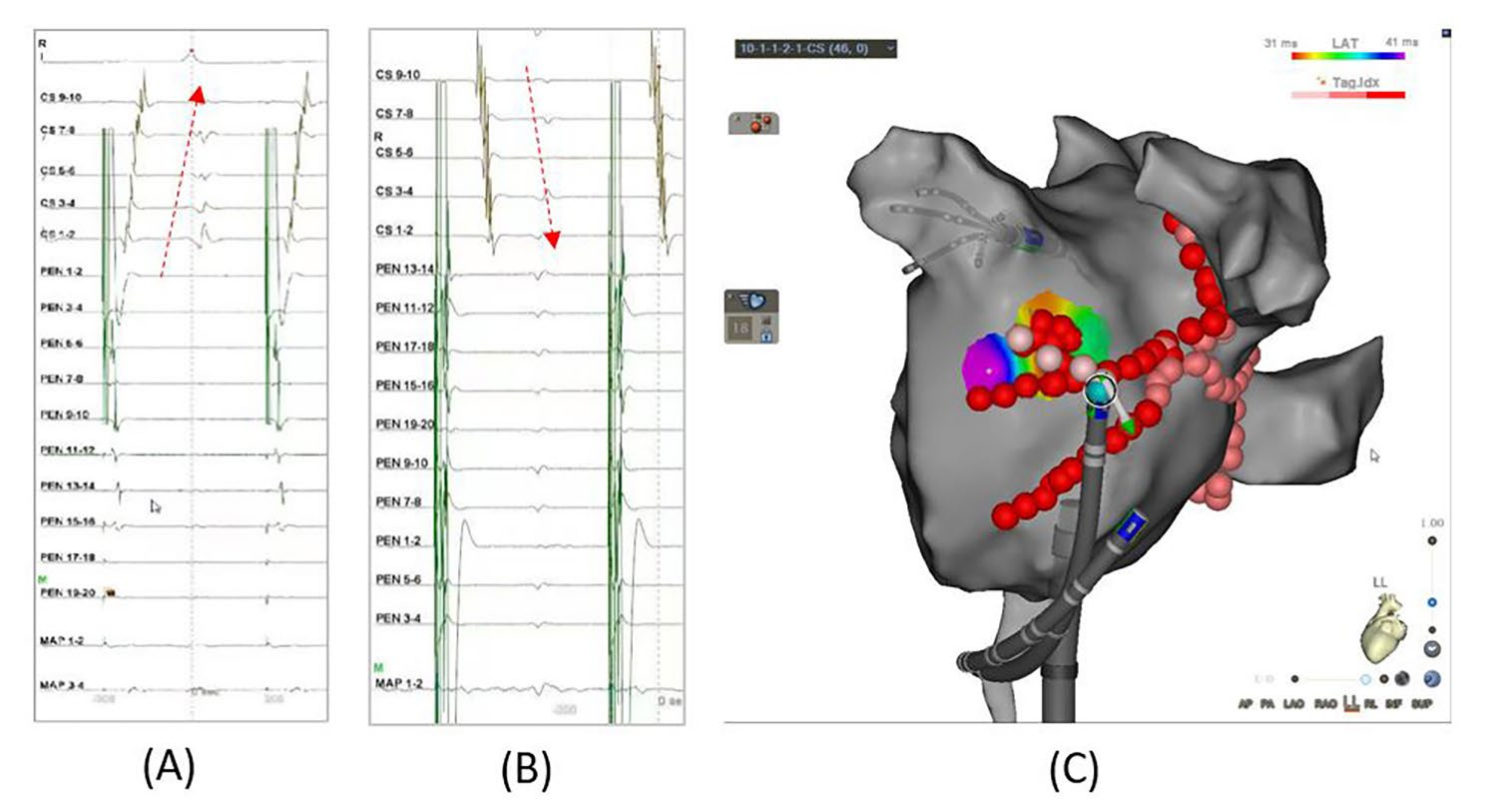
Precise epicardial ablation in the coronary sinus(CS). (**A**) The activation sequence was from distal to proximal CS catheter before epicardial ablation. (**B**) The activation sequence reversed after precise epicardial ablation pointing to the endocardial earliest activation sites (EASs) anatomically in CS.

An ablation catheter was inserted into the CS to perform precise epicardial ablation. ELs, EASs, and VOM ostium were defined as key ablation targets (KATs). The catheter was rotated and directed anatomically to the endocardial ELs and EASs for further ablation. In addition, extensive ablation was performed at the ostium of VOM. If the endpoint was not achieved, the procedure was terminated, and MI block failure was established (Fig. 6).

The effectiveness outcome was determined based on the presence of a bidirectional conduction block of MI after a 20 min waiting period, and complications related to the procedure were recorded. In addition, the block rate and operation time of every step was calculated.

### Statistical analysis

All statistical analyses were conducted with SPSS 25.0 software (SPSS Inc.). Continuous variables were expressed by the mean ± the standard deviation (SD) for normally distributed variables. Median (Q1, Q3) was used to express measurement data with a skewed distribution. The success rate of each step was presented as *n*(%).

## Results

### Baseline characteristics

A total of 135 PeAF patients underwent catheter ablation from March 2022 to March 2023. After excluding without visible VOM (n = 14, 10.4%) and cases of irreversible AF (n = 2), a final cohort of 119 patients underwent the stepwise catheter ablation method. The basic characteristics of these patients are summarized as follows in Table 1: The study cohort exhibited a male predominance (52.9%, n = 79) with a mean age of 60.8 ± 10.2 years and a median duration of AF of 24 (15, 32) months. The CHA2DS2-VASc score was 2.0 (1.0, 3.0). 14 (11.8%) patients had coronary artery disease (CAD), and 8 (6.7%) had a history of previous PVI. The average diameter of the LA was 44.8 ± 4.7 mm, and the average left ventricular ejection fraction (LVEF) was 53.5 ± 3.7%.

**Table 1:** Basic clinic characteristics of the patients (n = 119)

Characteristics	Value
Age, years (y, mean ± SD)	60.8 ± 10.2
Female, *n* (%)	56(47.1%)
CHF, *n* (%)	21(17.6%)
Hypertension, *n* (%)	46(38.7%)
Diabetes, *n* (%)	23(19.3%)
Stroke or TIA, *n* (%)	10(8.4%)
Vascular disease	9(7.6%)
CHA_2_DS_2_-VASc Score [median(IQR)]	2(1, 3)
CAD, *n* (%)	14(11.8%)
Prior PVI, *n* (%)	8(6.7%)
LVEF (%, mean ± SD)	53.5 ± 3.7
LA diameter(mm, mean ± SD)	44.8 ± 4.7
Duration of AF[mo., median(IQR)]	24(15, 32)

CHF: congestive heart failure; TIA: transient ischaemic attack; CHA_2_DS_2_VASc: Congestive heart failure, Hypertension, Age ≥ 75 years, Diabetes mellitus, Stroke/transient ischemic attack/thromboembolism, Vascular disease, Age 65 to 74 years, Sex Category; CAD: coronary artery disease; PVI: pulmonary vein isolation; LVEF: left ventricular ejection fraction; LA: left atrium; AF: atrial fibrillation; SD: standard deviation; IQR: interquartile range

### Stepwise procedure

As depicted in Table 2, None of the 119 patients who underwent the EI-VOM procedure (step 1) in an operation time of 10 (9, 10) minutes achieved a conduction block. Subsequently, the “V-shape” linear ablation (step 2) was performed in 119 patients. The operation time of this step was 13 (11, 14) minutes. Among them, 69 patients (58.0%) achieved bidirectional conduction block within the MI; 31 out of 69 (44.9%) achieved conduction block after the upper ablation line and the remaining (n = 38, 55.1%) after completion of the two lines. There were 227 ELs recorded in total. Of them, 133 out of 227 (58.6%) were located near the ostium of VOM, 51 out of 227 (22.5%) were located at the root of LAA, and the rest 43 out of 227 (18.9%) were distributed over the other parts of the two lines. In step 3, 50 patients underwent mapping of EASs and endocardial reinforced ablation in an operation time of 7 (6, 8) minutes. 22 out of 50 cases (44%) achieved conduction block, and the accumulated block rate was 76.5%. EASs that led to successful conduction block after ablation were mainly distributed at the root of LAA (n = 8, 36.4%) and the ostium of VOM (n = 10, 45.5%). The rest (n = 4, 18.2%) were located in the middle region between two lines of the “V-shape” ablation. Step 4 was initiated in the remaining 28 patients. It took 8 (7, 9) minutes to perform this procedure, and the block rate of this step was 92.9% (n = 26). Finally, a bidirectional conduction block of MI was achieved in 117 patients(98.3%). The fluoroscopy time was 5 (4, 7) minutes and was mainly spent on step 1. There was a significantly longer operation time (p < 0.001) needed to undergo all the steps of the ablation approach compared with that of achieving the conduction block at step 2.

**Table 2:** The time and effect of each step.(n = 119)

Data	Value
Total MI ablation time [min, median(IQR)]	26(23, 34)
Fluoroscopy time [min, median(IQR)]	5(4, 7)
Step 1(n)	119
Operation time[min, median(IQR)]	10(9, 10)
Conduction block	0(0%)
Step 2(n)	119
Operation time[min, median(IQR)]	13(11, 14)
Accumulated operation time[min, median(IQR)]	20(19, 21)
Conduction block	69(58.0%)
Accumulated block rate	58.0%
Step 3(n)	50
Operation time[min, median(IQR)]	7(6, 8)
Accumulated operation time[min, median(IQR)]	28(26, 30)
Conduction block	22(44%)
Accumulated block rate	76.5%
Step 4(n)	28
Operation time[min, median(IQR)]	8(7, 9)
Accumulated operation time[min, median(IQR)]	36(32, 37)
Conduction block	26(92.9%)
Accumulated block rate	98.3%

MI: mitral isthmus; IQR: interquartile range

### Complication

Four (3.4%) patients experienced complications in this study, including pericardial effusions (n = 2, no patient required pericardiocentesis), femoral hematoma (n = 1), femoral pseudoaneurysm (n = 1), and heart failure after the procedure (n = 1).

## Discussion

The present study introduced and evaluated a novel method of catheter ablation of MI involving EI-VOM, “V-shape” linear ablation, and endocardial and epicardial ablation of key ablation targets (KATs), abbreviated as “MVK”. Our main findings were as follows: (1) The implementation of the novel “V-shape” linear ablation technique yielded a positive impact on achieving MI block; (2) Precise ablation of KATs reduced unnecessary lesions, thereby minimizing the risk of complications; (3) The stepwise method contributed to a high bidirectional conduction block rate across the MI region; (4) The integration of well-established techniques in the stepwise method making it replicable by various cardiac electrophysiologists.

### Challenge of MI block

The mitral isthmus is an anatomic area between the mitral annulus and antrum of LPV that plays an important role in maintaining perimitral atrial arrhythmia [12, 13]. MI linear ablation is now recommended as an initial or repeat ablation strategy for PeAF [1]. However, achieving a bidirectional conduction block is challenging due to its complex anatomical structure. Detailed anatomy reveals that MI was surrounded by various structures, including LAA, LPV, CS, ligament of Marshall (LOM), and ramus circumflexus, as well as being inserted by some fat pads [14]. This anatomical complexity introduces several considerations when performing MI ablation. Firstly, there is a significant variation in myocardium thickness along the MI. Secondly, the ramus circumflexus and CS, two epicardial blood veins, serve as an epicardial “heat-sink” that prevents the formation of transmural lesions by lowering the conductive heating. Thirdly, autopsy studies revealed various numbers and morphologies of myocardium around the CS and/or LOM connecting with LA and serving as an endocardial-epicardial bridge across the MI line [5, 7, 14]. These difficulties pose challenges in creating transmural and durable lesions solely through endocardial ablation.

### Efficacy of EI-VOM

The VOM follows a posterior and superior course over the epicardial surface of the LA before draining into the CS. It forms an epicardial bridge consisting of VOM myofibers that can traverse the MI region from the CS to the ridge between the LPVs and LAA [15]. The presence of epicardial connections, especially those involving the VOM, can bypass the ablation lesions created in the MI area, resulting in an incomplete conduction block despite multiple radiofrequency applications [16, 17]. It is widely acknowledged that the selective ablation of EI-VOM targets the atrial myocardium supplied by the VOM [18]. By eliminating ectopic triggers originating from the VOM and blocking residual conduction mediated by epicardial connections, EI-VOM not only addresses the specific anatomic characteristics of the VOM but also extends its impact to the anterior wall of the LPV and the ridge between the LAA and LPV [17]. Accordingly, this enhances the durability of pulmonary vein isolation and modifies the substrate in the MI region [19]. In a recent study, Valderrabano et al. found that EI-VOM combined with RF catheter ablation superior to RF ablation alone improved the success rate of AF freedom and resulted in MI conduction block [8]. EI-VOM has now become widely accepted as a step in MI ablation procedures. Therefore, EI-VOM was selected as the first step of MI ablation for our approach. Nevertheless, despite previous demonstrations of its effectiveness, several pitfalls are associated with EI-VOM. Firstly, the chemical ablation of EI-VOM may not be capable of creating transmural lesions that cover the entire MI region. Secondly, the precision of the injury caused by EI-VOM can be challenging, and visible VOM is only present in a percentage of cases, ranging from 84 to 92% [8–10]. Thirdly, there were other epicardial connections except for VOM, which served as important residual MI gaps [20]. These pitfalls associated with EI-VOM accounted for the 0% block rate observed in the present study.

### “V-shape” endocardial linear ablation of MI

Endocardial linear ablation of MI is the most important step in achieving a conduction block. The conventional method defined the narrow region between LIPV and MA as the ablation line, generally originating from the root of LIPV to 2–3 o’clock of MA [12]. However, this method often resulted in a low acute block rate and high reconnection rate in the later stages of ablation due to MI’s heterogeneous and complex structure. In our study, a “V-shape” two-line design of endocardial ablation was selected. This method was inspired by previous ablation techniques for AFL related to the cavotricuspid isthmus(CTI) in the early years [21] and an ablation description of the epicardial connection of CTI in one study [22]. Multiple ablation lines were created to encompass all potential arrhythmogenic sites that were difficult to eliminate directly. In the present study, the upper line of “V- shape” ablation lines extended along the posterior root of LAA from the 1–2 o’clock point of the MA to LIPV while the lower was from the LIPV to the ostium of VOM. There were two advantages to this approach follows. Firstly, the ablation line was positioned away from the thick myocardium and the complex structure of the MI to some extent. Secondly, the “V-shape” block lines encompassed the residual epicardial connections after the initial EI-VOM ablation. These findings account for the higher acute block rate of MI (57.9%) observed in our study compared to the literature.

### Endocardial and epicardial ablation of KATs

Due to the complex structure of the MI and variations in myocardium thickness, different RF deliveries are needed to achieve durable lesions. In the present study, ELs were described as lesions where the activation sequence changed or the conduction time was prolonged during the ablation process, indicating that myocardial conduction passed through those sites. Enhanced ablation of these ELs improved the acute success rate of MI conduction block and reduced the likelihood of late reconnection. Mapping and ablation of EASs were performed when the initial linear ablation did not achieve a conduction block, which helped circumvent the need for extensive additional ablation [20, 23]. Chen et al. reported that conduction breakthroughs were observed in 78.4% of patients after the initial linear ablation [23]. In our study, residual connections were identified in 50 patients(42.0%), which may be attributed to the early EI-VOM, which eliminated some epicardial connections. Endocardial ablation of EASs could facilitate conduction block in 22 cases, primarily at the root of LAA(36.4%) and the ostium of VOM(45.5%), possibly due to thicker myocardium or elimination of some epicardial connections at the two positions. Ablation of VOM ostium was performed due to the incomplete alcoholization caused by balloon occlusion [24]. The remaining 28 patients needed epicardial ablation to achieve a conduction block, attributed to the residual epicardial connections. Precise endocardial and epicardial ablation of these connections reduced the need for excessive ablation and decreased the recurrence of complications, such as cardiac tamponade.

Herein, three techniques were involved: EI-VOM, endocardial linear RF ablation, and endocardial and epicardial ablation of KATs. These methods have been extensively utilized over the years, and their effectiveness and feasibility have been well-documented in prior research. Therefore, there are no technical barriers constraining the practicability of this gradual procedure, making it replicable by various cardiac electrophysiologists. Moreover, advances in technologies, including contact force (CF)-sensing catheters, high-power ablation strategy, and visualized steerable sheaths, facilitated the creation of contiguous lesions by resistive heating, which was beneficial in minimizing the likelihood of late reconnections [25–27].

### Limitation

Firstly, notwithstanding the promising outcomes of our investigation, it is imperative to recognize its constraints, which encompass a limited sample size derived from a solitary center and the absence of a control group. Secondly, the present work has not specifically evaluated the long-term outcomes. Our research group followed all patients in our study, and the follow-up results will be published in future studies, providing insights into the short and long-term prognosis.

## Conclusion

Our innovative stepwise catheter ablation technique for MI incorporated well-established methods and demonstrated a consistently high rate of bidirectional block, replicable by various cardiac electrophysiologists.

## Data Availability

Shujuan Dong could be contacted if someone wants to request the data from this study.
